# Characterization of Aroma-Active Compounds in Five Dry-Cured Hams Based on Electronic Nose and GC-MS-Olfactometry Combined with Odor Description, Intensity, and Hedonic Assessment

**DOI:** 10.3390/foods14132305

**Published:** 2025-06-29

**Authors:** Dongbing Yu, Yu Gu

**Affiliations:** School of Biomedical Engineering, Capital Medical University, No. 10, Xitoutiao, YouAnMen, Fengtai District, Beijing 100069, China; yudongbing@mail.ccmu.edu.cn

**Keywords:** aroma-active, dry-cured hams, electronic nose, gas chromatography-olfactometry, hedonic assessment

## Abstract

The evaluation of aroma-active profiles in dry-cured hams is crucial for determining quality, flavor, consumer acceptance, and economic value. This study characterized the volatile compounds in five varieties of dry-cured hams using gas chromatography-mass spectrometry-olfactometry (GC-MS-O) and an electronic nose (E-Nose). In total, 78 volatile compounds were identified across five varieties of dry-cured hams. A total of 29 compounds were recognized as aroma-active compounds. Odor description, intensity, and hedonic assessment were employed to evaluate these compounds. Black Hoof Cured Ham and Special-grade Xuan-Zi Ham contained higher levels of favorable compounds such as nonanal, 5-butyldihydro-2(3H)-furanone, and 2,6-dimethylpyrazine, contributing to sweet and popcorn-like notes. In contrast, Fei-Zhong-Wang Ham and Liang-Tou-Wu Ham exhibited higher proportions of off-odor compounds with lower hedonic scores. A principal component analysis clearly separated the five hams based on their aroma-active profiles, and a correlation analysis between E-Nose sensor responses and GC-MS-O data demonstrated a strong discriminatory ability for specific samples. These findings offer valuable insights into the chemical and sensory differentiation of dry-cured hams and provide a scientific basis for quality control, product development, and future improvements in E-Nose sensor design and intelligent aroma assessment.

## 1. Introduction

Dry-cured ham is a high-value meat product with significant consumer acceptance, and it is typically classified according to its region of origin [1]. The final flavor of dry-cured ham is influenced by unique factors, including climate, geography, pig breed, and production methods specific to each region [2]. For example, in the Mediterranean, Spanish Iberian ham is highly esteemed and produced from Iberian pigs that are fed acorns and pasture [3]. Moreover, among Chinese styles of dry-cured hams, Xuanwei ham, and Jinhua ham are highly valued due to their long history [4]. Quality differences among dry-cured hams are substantial, reflected in their pricing, with Iberian ham often being several times more expensive than other hams. However, the high commercial value of the dry-cured ham with geographical indications has led to increased labeling fraud concerning both the origin and grade of these products [5]. Therefore, comprehensive research into the flavor profiles of various dry-cured hams is essential to protect consumer interests and producers’ reputations.

Flavor, a key attribute influencing consumer acceptance and economic value, is largely determined by the distinct aroma of dry-cured ham [6]. The quality of dry-cured ham is traditionally evaluated by its aroma intensity and persistence using the “three sticks” method, where bamboo sticks are inserted into the sacral vertebra, knee joint, and hip joint of hams [7,8]. Aroma is thus a crucial sensory characteristic [9]. During the production process, dry-cured hams undergo various chemical and biological reactions, including protein hydrolysis, lipid degradation and oxidation, Maillard-type reactions, and Strecker degradation. These processes generate a complex array of volatile compounds that affect overall acceptance [7]. However, not all volatile compounds are aroma-active due to varying odor thresholds and interactions between compounds [10,11]. Some volatiles may exist at high concentrations but exert minimal olfactory impact due to their high detection thresholds. In contrast, aroma-active compounds (even at trace levels) can be detected by specialized olfactory cells and play a decisive role in defining product flavor [6]. Therefore, studies on the aroma-active compounds that contribute to the flavor are more important to the development of dry-cured hams.

In recent years, gas chromatography-mass spectrometry (GC-MS) and electronic nose (E-Nose) technologies have been extensively used to analyze the flavor profiles of dry-cured hams [12,13,14,15]. GC-MS is a widely utilized technique for detecting volatile components with high sensitivity and selectivity [16]. However, GC-MS alone can be insufficient for identifying aroma-active volatiles that contribute specifically to ham flavor. To address this limitation, a combination of GC-MS with olfactometry (GC-MS-O) has been employed to characterize aroma-active compounds. In this integrated technique, volatile analytes are first resolved on the GC column, after which the effluent is divided so that one fraction enters the mass spectrometer for structural elucidation while the other is simultaneously channeled to a sniffing port [17]. A panel of assessors typically sniffs the GC effluent to provide detailed aroma data. Although considerable research has focused on the aroma composition of dry-cured ham [2,10,18], studies utilizing GC-MS-O have predominately centered on describing odors by linking them to known aromatic substances. Beyond odor description, aromas can be quantified by additional dimensions such as intensity and hedonic assessment [19]. Intensity refers to the strength of the aroma sensation, while hedonic assessment evaluates the relative pleasantness or unpleasantness of the aroma. The preferred sensory attributes trigger “pleasant” emotions to consumers [20]. Therefore, GC-MS-O experiments incorporating more aroma dimensions are essential for a comprehensive understanding of dry-cured ham aroma.

The E-Nose is a chemical measurement system that detects volatiles through an array of gas sensors, generating a fingerprint response to the sample gases [21]. Recent advancements have promoted the E-Nose as a bionic device that simulates the mammalian olfactory system [22]. This has spurred interest in using the E-Nose as a cost-effective alternative to human ‘sniffers’. However, in 2014, Peter Boeker proposed that the E-Nose is not fully effective for measuring odor qualities [23]. Although it mimics the biological structure of olfaction, the gas sensors of a classical E-Nose are weakly selective and lack a specific filter function for odorants similar to odor receptor cells. Moreover, unspecific gas sensors are not odor sensors but react on volatiles regardless of whether they are aroma-active compounds. Therefore, the currently designed E-Nose is limited in its effectiveness for precise odor measurement.

This study aims to identify the aroma-active compounds in five different dry-cured hams using a combination of GC-MS-O and E-Nose technologies. The GC-MS-O experiments evaluated the odor description, intensity, and hedonic assessment of the aroma-active compounds. The principal component analysis (PCA) method was used in conjunction with GC-MS-O to differentiate odor attributes among the hams. Additionally, correlations between the GC-MS-O results and E-Nose responses were analyzed to enhance understanding of how these technologies relate to the human perception of odor. This work offers new insights into designing E-Nose systems that better replicate the biological structure of the sense of smell.

## 2. Materials and Methods

### 2.1. Ham Samples

**Figure 1:**
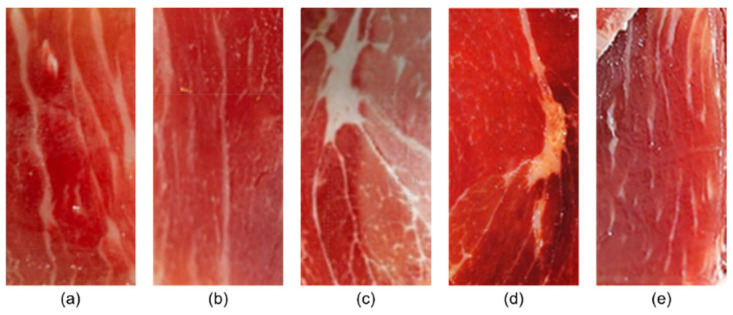
Photos of the five dry-cured hams. (**a**) BHCH; (**b**) SXZH; (**c**) FXZH; (**d**) LTWH; (**e**) FZWH.

Five ham samples from renowned categories (one from Bama Ham, two from Xuanwei Ham, and two from Jinhua Ham) were purchased from specialty stores authorized by the China Meat Association in Beijing (October 2024), including Black Hoof Cured Ham (BHCH), Superior-grade Xuan-Zi Ham (SXZH), First-grade Xuan-Zi Ham (FXZH), Liang-Tou-Wu Ham (LTWH), and Fei-Zhong-Wang Ham (FZWH). For Bama Ham (BHCH), Spanish white pig hind legs were first hand-rubbed with coarse Mediterranean Sea salt (7% *w*/*w*) and lightly massaged every 24 h for 10 days, then air-dried for 60 days in a temperature- and humidity-controlled chamber (4–6 °C, 75% RH). Subsequent maturation was carried out in an underground cellar (12–18 °C, 65% RH) for 12 months, with periodic greasing to prevent surface fissures. Manufacturer data list moisture 45%, protein 25%, fat 20%, and salt 5% (g 100 g^−1^). Both Xuanwei hams (SXZH and FXZH) were produced from “Highland Wujin” pigs and subjected to the traditional three-stage dry-salting procedure (2.5%, 3.0%, and 1.5% of leg weight at 3 d intervals). SXZH was matured for 36 months, qualifying as special grade according to the local quality standard—a characteristic “three-pin aroma”. FXZH followed the same technique but was marketed after ≥ 24 months of aging, meeting first-grade requirements. Typical proximate values for mature Xuanwei hams are moisture 40–42%, protein 26–27%, fat 23–25% and salt ≈ 4%. The traditional processing of Jinhua Ham uses the indigenous “Two-Ends-Black” pig breed; modern factories additionally process cross-breeds. Fresh legs are salted with 6–8% NaCl for 30–40 days, washed, sun-dried, and mold-ripened in ventilated lofts before a final piling stage at 15–18 °C. LTWH declares the use of Two-Ends-Black pigs and air-drying followed by 24 months of cellar aging. FZWH employs free-range local pigs and a 12-month maturation. After ripening, Jinhua ham typically contains moisture 38–40%, protein 27–28%, fat 22–23%, and salt 5%. The details of the ham samples are provided in Table 1, and their photographs are shown in Figure 1. All samples were stored in their original containers at 2 °C in a laboratory refrigerator until analysis.

### 2.2. HS-SPME-GC-MS-O Analysis

**Figure 2:**
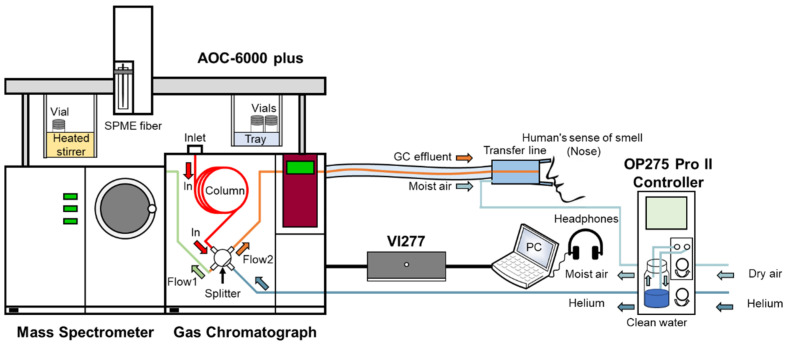
Schematic diagram of the GC-MS-O workflow.

Volatile compounds were extracted using headspace solid-phase microextraction (HS-SPME) with a multifunctional autosampler (AOC-6000 plus, Shimadzu, Kyoto, Japan). Three independent extractions were carried out for each ham sample. Identification of the volatile compounds was performed using gas chromatography-mass spectrometry (GC-MS-TQ8040 NX, Shimadzu, Kyoto, Japan). As shown in Figure 2, prior to extraction, a smart SPME fiber (DVB/Carbon WR/PDMS, 80 µm, Shimadzu, Muttenz, Switzerland) was conditioned for 30 min at 250 °C in the gas chromatograph injection port (Inlet). One gram of minced ham sample, with 5 µL of an aqueous solution containing 53.4 mg/L of cyclohexanone (GC quality, Aladdin, Shanghai, China) as an internal standard, was placed in a 20 mL headspace vial and sealed with a screw cap. The compounds were extracted at 60 °C for 60 min in a heated stirrer after equilibrating at 60 °C for 2 min. The adsorbed compounds were then desorbed in the GC injector for 30 min at 250 °C. Volatile compounds were separated using an SH-Rxi-5Sil MS capillary column (0.25 mm × 0.25 µm × 30 m, Shimadzu, USA). Helium was used as the carrier gas with a column flow rate of 2 mL/min. The chromatograph oven temperature was as follows: 40 °C for 1 min, increased from 40 °C to 100 °C at 3 °C/min, held at 100 °C for 10 min, increased from 100 °C to 150 °C at 5 °C/min, held at 150 °C for 2 min, and finally increased from 150 °C to 250 °C at 10 °C /min with a 5 min hold. The MS operated in Q3 Scan mode over a range of *m*/*z* 33–500. Compounds were identified by comparing their mass spectra with the NIST20s mass spectral library. The concentration of each compound was calculated based on its area relative to the internal standard.

Odor-active compounds were characterized using a sniffing port (OP275 Pro II, GL Sciences, Tokyo, Japan) coupled with the GC system. As depicted in Figure 2, the samples separated by the capillary column were split at the column outlet. One branch (Flow 1) was directed to a gas chromatograph equipped with a mass-spectrometry detector for structural identification, while the other branch (Flow 2) was carried away from the gas chromatograph by auxiliary gas (Helium) to a sniffing port and evaluated using a human’s sense of smell (nose). To prevent nasal mucosa drying and to mitigate its negative effect on sensory perception during prolonged GC-MS-O analysis, the moist air generated in a bubbling bottle was introduced at the nose cone flange. The GC-MS conditions were consistent with those previously described. An olfactory voicegram device, consisting of an olfactory voicegram interface (VI277, GL Sciences, Tokyo, Japan) and a set of USB headphones, was used to record the names and details of odors alongside the chromatogram.

A ten-member panel of assessors, comprising both sexes and aged between 20 and 30 years, was recruited for the GC–O analyses. The panelists were trained prior to the sensory evaluation to become familiar with odor descriptions. During the analysis, each panelist individually sniffed the GC effluent and reported their observations. The aroma-active compounds detected were recorded along with their perceived intensity, quality, and hedonic assessment. Intensity, representing the strength of the aroma sensation, was classified into three levels: strong, middle, and weak. Aroma quality was described using standard descriptors, which are common terms that relate the aroma to known substances [19]. The aroma quality standards used in the experiments are shown in Table 2, including 10 aroma groups with examples of descriptor types. The hedonic assessment dimension was associated with the relative pleasantness or unpleasantness of the aroma and quantified on a scale from 1 (completely dislike) to 10 (very good, pleasant, and agreeable). Volatile compounds detected by more than two panelists were considered to have potential aroma activity [10].

### 2.3. E-Nose Analysis

**Figure 3:**
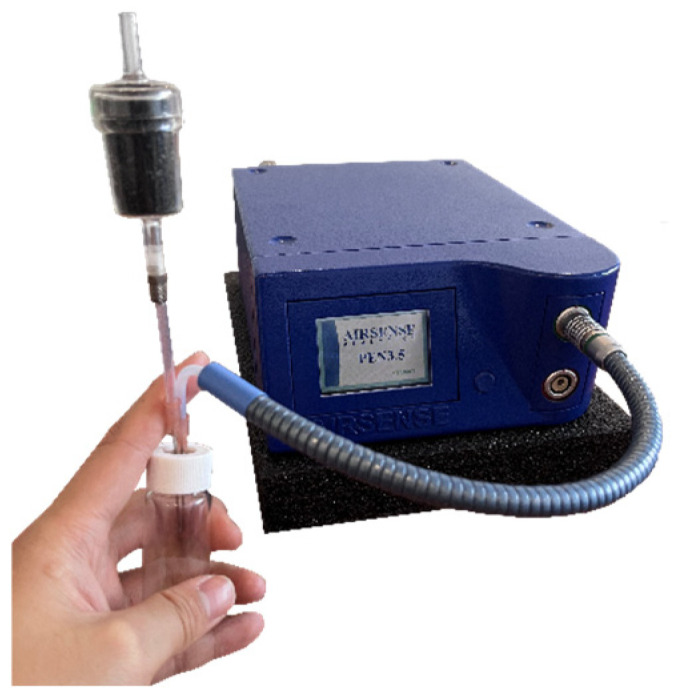
Photo of the PEN3 E-nose.

The E-Nose analysis was conducted using a commercial E-Nose (PEN3, Airsense Analytics GmbH, Schwerin, Germany, Figure 3) with 10-MOS sensors to acquire the volatile compound profile of the ham samples. The details of the MOS sensors are shown in Table 3 [24].

**Figure 4:**
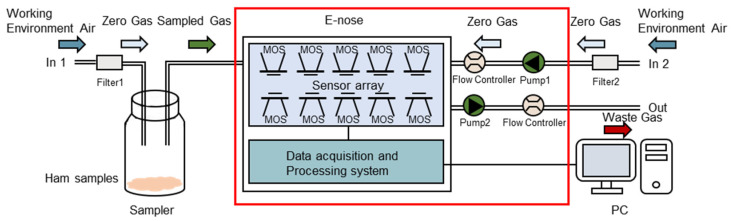
Schematic diagram of the PEN3 workflow.

The experiments were conducted for 12 days in a clean, well-ventilated testing room (approximately 45 square meters) at a temperature of 25 °C ± 1 °C and a humidity level of 40% ± 2%. Each of the five ham samples was measured 10 times per day by a skilled operator, with fresh samples used for each daily experiment. To minimize baseline fluctuations and interference from other volatiles, volatile compound profiles were acquired in a controlled environment. Zero gas (a baseline) was generated using two active charcoal filters (Filter 1 and Filter 2 in Figure 4) to ensure consistency between reference air and sample air.

The E-Nose analysis comprised two stages: collection and flushing. As illustrated in Figure 4 [24], one gram of the ham sample was placed into a 20 mL sampler for 180 s to allow the volatile compounds to disperse into the sampler. Before the collection stage, clean air was directed into the device in the direction of In 2 (flow rate: 10 mL/s, time: 100s) for automatic adjustment and calibration. This process is known as zero-point trim. Values relative to the zero-point values were recorded as a baseline. Following the zero-point trim, the volatile gases from the sampler were pumped into the device in the direction of In 2 (flow rate: 10 mL/s, time: 100 s). The conductivity of the E-Nose sensors changed in response to gas molecule absorption and stabilized at a constant value once adsorption was saturated. During the collection stage, the sampling continued at one sample per second and lasted for 100 s. The sensor chamber was flushed between measurements. In the flushing stage, clean air was pumped into the device in the direction of In 2 (flow rate: 10 mL/s, time: 100s) to remove residual analytes. This collection and flushing process was repeated to obtain the raw data of the five ham samples.

### 2.4. Statistical Analysis

All the HS-SPME-GC-MS analyses were conducted in triplicate for each ham sample, and the results were presented as means ± standard deviation (SD). One-way analysis of variance (ANOVA) was conducted using OriginPro 2025 to assess the significant differences in volatile compound concentrations among sample groups. A Venn diagram, principal component analysis (PCA), and heatmap were also performed by means of the OriginPro 2025.

The Venn diagram was employed to visualize the distribution of volatile compounds among the sample groups, facilitating the identification of both shared and unique compounds. This graphical representation aids in understanding the compositional overlap and specificity among different samples.

The aims of the PCA were to reduce the dimensionality of the dataset and to identify underlying patterns in the volatile compound profiles. The PCA transforms the original variables into a new set of uncorrelated variables (principal components), which capture the maximum variance in the data, thereby facilitating the visualization of sample clustering and the detection of latent variables.

The heatmap was used to depict the relationships between volatile compounds and sample groups. This visualization technique allows for the assessment of similarities and differences among samples based on their volatile profiles, providing insights into the correlation patterns and aiding in the interpretation of complex datasets.

## 3. Results and Discussion

### 3.1. Volatile Compounds Analysis

**Figure 5:**
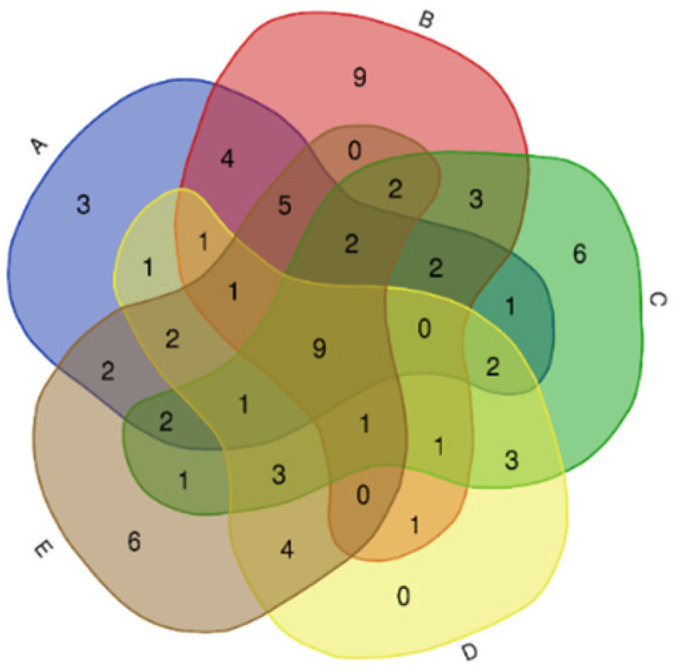
Venn diagram of volatile compounds in five different types of ham samples. A: BHCH, B: SXZH, C: FXZH, D: LTWH, E: FZWH.

The volatile compounds identified in the five different dry-cured hams (BMCH, SXZH, FXZH, LTWH, and FZWH) using HS-SPME-GC-MS-O are listed in Table 4. A total of 78 volatile compounds were identified and semi-quantified, and these were classified into eight chemical families: alcohols, aldehydes, alkanes, ketones, esters, acids, terpenes, and others. As shown in Table 4, the number of identified volatile compounds varied among the samples: 38 for BMCH, 41 for SXZH, 39 for FXZH, 30 for LTWH, and 41 for FZWH. SXZH and FZWH had the highest number of identified volatile compounds, while LTWH and FZWH exhibited the greatest variety of compounds. A Venn diagram based on the profiles of volatile compounds across the different ham samples is shown in Figure 5. A total of nine volatile compounds were identified in all five ham samples, namely nonanal (B3), benzaldehyde (B4), phenylethyl alcohol (A8), 2,6-dimethylpyrazine (H1), phenylacetaldehyde (B7), dodecane (C3), dihydro-5-pentyl-2(3H)-furanone (D6), 5-butyldihydro-2(3H)-furanone (D8), and tetradecane (C5). Among the nine volatile compounds, nonanal (B3) and benzaldehyde (B4) were the most abundant overall. These compounds are typically generated via the β-oxidation and autoxidation of unsaturated fatty acids during the early stages of drying and throughout the cellar-aging process [25]. Nonanal (B3) and benzaldehyde (B4) showed particularly high concentrations in both SXZH and FZWH, indicating more extensive lipid oxidation in these samples due to their longer maturation times or higher fat degradation levels. Phenylethyl alcohol (A8), a secondary alcohol often associated with amino acid metabolism or the further oxidation of aldehydes [26], was found in significantly higher concentrations in SXZH than in other samples. This may reflect either prolonged proteolysis or deeper oxidative transformation pathways during the longer maturation periods of the products. Specific volatile compounds were unique to each ham type: three to BHCH, nine to SXZH, six to FXZH, and six to FZWH. SXZH had the largest number of specific volatile compounds, while LTWH possessed no specific volatile compounds. These unique volatile compounds may originate from distinct differences in raw materials, microbial flora, and processing conditions. Volatile compounds (i.e., 2,4-dimethyl-heptane, 3-octanone, and (E)-2-heptenal) were identified only in BHCH. The exclusive presence of 2,4-dimethyl-heptane and (E)-2-heptenal in BHCH could be associated with the oxidative degradation of unsaturated fatty acids under Spanish-style cellar-aging at lower humidity, which favors lipid autoxidation and long-chain alkane fragmentation [27]. Volatile compounds such as 2-pentyl-furan, 2,4-dimethyl-hexane, 2-nonanol, 2-tetradecanone, tridecanal, nonanoic acid ethyl ester, 1-tetradecanol, amyl cyclopentenone, and acetic acid hexyl ester were identified only in SXZH. The higher number of unique volatiles in SXZH may reflect its extended 36-month cave maturation and distinct microbial enzymatic activities, which are known to catalyze esterification and alcohol formation. Moreover, hexanoic acid ethyl ester, 3,5-octadien-2-one, 2,2,4,4,6,8,8-heptamethyl-nonane, 3,7-dimethyl-1-octanol, tetramethyl-pyrazine, and dimethyl trisulfide were identified only in FXZH. Tetramethyl-pyrazine and dimethyl trisulfide are likely linked to Maillard reaction pathways and sulfur amino acid catabolism during protein degradation under prolonged ripening. Volatile compounds such as 3-octen-2-one, acetic acid, ethanol, (E)-2-octen-1-ol, cis-4-decenal, and (1S, cis)-4,7-dimethyl-1,2,3,5,6,8a-hexahydro-naphthalene were identified only in FZWH. Previous studies have shown that the overall odor quality of hams can be characterized by several volatile compounds (Jiang et al., 2022) [2]. Therefore, the specific volatile compounds contribute to the distinct characteristics and flavor profiles of the different dry-cured hams.

Fifteen alcohols were detected across the samples. Alcohols, primarily produced through lipid oxidation [28], play a crucial role in ham flavor, contributing herbaceous, woody, and fatty notes [29]. Alcohols with sweet, fruity, or onion and mushroom-like odors are notable in this group [30]. Phenylethyl alcohol (A8), found in all samples, is significant for its aromatic floral and fruity notes [31]. Research indicates that phenylethyl alcohol (A8) levels were low at three months during the ripening period, but increased after 12 months [1,31,32], which could explain its varying levels among the samples. In this study, SXZH, with a 36-month maturation period, exhibited the highest concentration of phenylethyl alcohol (A8) (875.16 ± 2.92 ng/g), aligning with previous findings that extended ripening enhances the accumulation of such compounds. BHCH, despite a shorter maturation, showed relatively high levels (337.77 ± 2.71 ng/g), which may be attributed to its specific processing conditions, such as controlled temperature and humidity during cellar aging, potentially accelerating proteolysis and the subsequent formation of phenylethyl alcohol (A8) [33,34]. Compounds like 1-octen-3-ol (A3) and 1-octanol (A7) have low odor thresholds and significantly impact the aroma profile [35]. Herein, 1-octen-3-ol (A3), known for its strong mushroom aroma, was the most abundant alcohol detected in FZWH (4414.67 ± 13.68 ng/g) and is commonly found in meat products such as Istrian dry-cured ham [1]. 1-Octanol (A7), which imparts fatty, sharp aroma notes [35], was present in BHCH, SXZH, and FZWH. Benzyl alcohol (A9), contributing floral notes [36], was detected in BHCH, LTWH, and FZWH. Notably, BHCH and FZWH, the most expensive and least expensive hams, respectively, shared nine alcohols, excluding 2-heptanol (A1), ethanol (A14), and (E)-2-octen-1-ol (A15). 2-Heptanol (A1) was unique to BHCH and was the most abundant alcohol in this sample (1816.45 ± 5.39 ng/g). Ethanol (A14) and (E)-2-octen-1-ol (A15) were only present in FZWH, albeit in low concentrations (359.18 ± 3.18 ng/g and 217.8±3.43 ng/g, respectively). As reported in previous reports, ethanol (A14) is not significantly known for its aroma [37]. Thus, 2-heptanol (A1) likely plays a key role in differentiating BHCH from FZWH. SXZH, the second most expensive ham, had seven alcohols, which were also detected in BHCH. The unique presence of 2-Nonanol (A10) in SXZH may aid in the identification of SXZH among the five hams. Conversely, 1-octen-3-ol (A3), one of the alcohols with low thresholds, was detected in BHCH but not in SXZH, suggesting it may be a distinguishing factor between BHCH and SXZH.

Among the detected volatile compounds, aldehydes were the most abundant group, with eighteen aldehydes identified in this study. Aldehydes are significant degradation products of lipid oxidation, but they can also arise from Maillard-induced amino acid degradation [38]. Aldehydes play an important role in the overall flavor of dry-cured hams because of their low odor thresholds, high concentrations, and distinctive characteristic odors (e.g., sweet, floral, pungent notes) [2,31,39,40]. Among the aldehydes, nonanal (B3), benzaldehyde (B4), and phenylacetaldehyde (B7) were present in all ham samples. Nonanal (B3), a common volatile compound in meat products, is a linear-chain aldehyde that contributes to sweet and fruity aromas [41]. Other typical linear-chain aldehydes, such as hexanal (B1), octanal (B2), decanal (B13), and heptanal (B17) were also detected in the ham samples. These linear aldehydes are mainly generated from the degradation of long-chain fatty acids [42,43]. Hexanal (B1), found in BHCH, FXZH, LTWH, and FZWH, was the most abundant aldehyde among the four hams (7859.9 ± 19.9 ng/g, 3310.9 ± 6.49 ng/g, 638.9 ± 0.67 ng/g, and 13283.48 ± 29.52 ng/g, respectively). In general, hexanal (B1) is known as a major oxidation product in dry-cured meats and serves as a good indicator of oxidation levels [18,44,45]. The high concentrations of hexanal (B1) observed align with the findings from studies on Istrian and Iberian hams [38,46]. Octanal (B2) was detected in BHCH, LTWH, and FZWH but not in Xuanwei hams (SXZH and FXZH), likely due to differences in spice additions during the manufacturing process. Another important aldehyde detected in the dry-cured ham was the branched-chain saturated aldehyde, resulting from the Strecker degradation of amino acids like valine and leucine [7,40,47], including benzaldehyde (B4), phenylacetaldehyde (B7), and 3-methylbutanal (B16). These aldehydes increase during fermentation and contribute green, honey, fatty, and chocolate-like odors, enhancing the meaty odor and reducing sour notes [40,48]. 3-Methylbutanal (B16), a major contributor to Istrian dry-cured ham flavor [49,50], was found in FXZH, LTWH, and FZWH.

Alkanes are usually formed by the oxidation of unsaturated fatty acids [2]. Herein, 14 alkanes were detected. Alkanes generally contribute minimally to the overall flavor of dry-cured hams due to their high thresholds [6,35]. In this study, the concentration of the detected alkanes was relatively low compared to the major compound groups like alcohols and aldehydes. Notably, BHCH exhibited a significantly higher concentration of 2,4-dimethyl-heptane (C1) at 2940.23 ± 14.08 ng/g, which was not detected in the other samples. This suggests a unique lipid oxidation pathway or raw material characteristic specific to BHCH. Similarly, SXZH showed a markedly elevated level of 2,4-dimethyl-hexane (C7) at 4748.34 ± 18.47 ng/g, indicating a distinct oxidative process possibly influenced by its extended maturation period of 36 months. The presence and concentration of these alkanes can be influenced by several factors, including the degree of lipid oxidation, the composition of fatty acids in the raw material, and the specific processing parameters such as temperature, humidity, and the duration of curing [51,52]. For instance, extended curing times and higher temperatures can enhance lipid oxidation, leading to an increased formation of alkanes. Although the alkanes are not primary contributors to the aroma due to their high odor thresholds, their presence serves as an indicator of the extent of lipid oxidation and can reflect the oxidative stability and quality of the ham. Therefore, monitoring the levels of specific alkanes can provide valuable insights into the processing conditions and potential shelf-life of dry-cured hams.

In this study, 15 ketones were detected, which are generally derived from lipid oxidation, amino acid degradation, and the Maillard reaction [38,53,54]. These compounds contribute to the fatty aromas associated with cooked meat [55,56]. Among the detected ketones, dihydro-5-pentyl-2(3H)-furanone (D6) and 5-butyldihydro-2(3H)-furanone (D8) were present in all ham samples at relatively low concentrations. 2-Heptanone (D1) and 2-nonanone (D2) were the most and second most abundant ketones detected in BHCH (1291.03 ± 3.03 ng/g and 975.78 ± 3.48 ng/g, respectively) and were also found in SXZH (979 ± 2.68 ng/g and 1166.89 ± 2.24 ng/g, respectively). These two ketones are also present in Istrian dry-cured hams [57]. Notably, 2-Heptanone (D1) contributes to the dry-cured ham aroma with spicy/blue cheese/acorn sensory notes, whereas 2-nonanone (D2) contributes to ham aroma with floral/fruity/blue cheese aroma. 3-Octanone (D3), unique to BHCH (839.6 ± 1.95 ng/g), likely originates from rosemary used in the production process and aligns with findings in Istrian dry-cured hams [6,58]. Acetophenone (D11) was detected in the two Xuanwei hams (187.88 ± 4.51 ng/g in SXZH and 70.65 ± 1.00 ng/g in FXZH), indicating its association with long-ripened raw hams [48] and distinguishing Xuanwei from non-Xuanwei hams.

Seven esters were detected, formed by the esterification of various acids and alcohols. Esters typically increase with a longer ripening process [31,39,59] and impart fruity and sweet notes to dry-cured hams. The contribution of esters to the overall aroma depends on the length of their chain [35,60,61]. Esters formed from short-chain acids provide fruity notes while long-chain acids impart fat odors [62]. The relatively low amounts of esters in this study may be attributed to the antimicrobial activity of sodium chloride during the long curing period [37,63]. Despite the overall low concentrations, substantial differences were observed among the ham types. SXZH exhibited the highest diversity and abundance of esters, including octanoic acid ethyl ester (E1) (499.38 ± 3.79 ng/g) and butanoic acid 3-methyl ethyl ester (E2) (370.83 ± 4.09 ng/g), which are known to impart fruity and fermented notes. FXZH also showed high levels of hexanoic acid ethyl ester (1207.78 ± 0.79 ng/g). In contrast, BHCH and LTWH presented no detectable esters, and only trace amounts were found in FZWH. These differences may reflect variations in microbial activity, enzymatic hydrolysis, and raw material properties among the products. In particular, the extended aging time and potentially more diverse microbial ecosystem of SXZH could favor the formation of esters [64,65]. Moreover, the absence of detectable esters in BHCH might be linked to its tightly controlled ripening environment and higher salt content, which can suppress microbial fermentation pathways essential for ester generation [66,67].

### 3.2. Analysis of Aroma-Active Volatiles by GC-MS-O

Although GC-MS identified the volatile compounds in the five different hams, it was unclear which compound truly contributed to the flavor of the hams. Therefore, GC-MS-O analysis was performed to identify the aroma-active compounds in the dry-cured hams. A ten-member panel assessed the intensity, quality, and hedonic aspects of the volatile compounds when perceiving the odor. The quality of these aroma-active compounds was confirmed by comparing them with descriptors from the 10 aroma groups listed in Table 5. Note that a single aroma-active compound could correspond to multiple aroma groups due to variations in odor at different concentrations. The odor intensity (OI) of each aroma-active compound was rated by the panelists, while the hedonic assessment was calculated as the average of the scaled values (1 to 10) provided by the panelists.

The GC-MS-O analysis results are summarized in Table 5. Of the 78 volatile compounds identified by GC-MS, 29 were classified as aroma-active compounds by the detection frequency analysis (DFA) method (detection frequency ≥ 2). Additionally, 10 unknown compounds that had concentrations below the GC-MS detection threshold were found but not specifically characterized. The GC-MS-O analysis revealed that the aroma-active compounds identified in dry-cured hams covered a broad range of aroma groups, including “woody/resinous”, “pungent”, “chemical”, “popcorn”, “fruit, other than citrus”, “lemon”, “minty”, “sweet”, “sickening”, and “floral”. Among these aroma groups, “woody/resinous”, “chemical”, “popcorn”, and “sweet” were the most frequently noted. Among these 29 aroma-active compounds, three compounds, namely nonanal (B3), 5-butyldihydro-2(3H)-furanone (D8), and 2,6-dimethylpyrazine (H1), were identified in all five ham samples. These three compounds contribute to the “sweet” and “popcorn” odor, which may generally influence the overall aroma of the hams. Nonanal (B3) is a major aldehyde derived from the oxidative degradation of unsaturated fatty acids, particularly oleic acid, during the curing and aging process. It has a low odor threshold, making it a critical compound in the perception of matured meat products [45]. 5-Butyldihydro-2(3H)-furanone (D8), belonging to the lactone/furanone family, likely forms via thermal degradation of carbohydrates or Maillard-type reactions involving reducing sugars and amino acids during long-term aging [68]. 2,6-Dimethylpyrazine (H1) is typically generated from Maillard reactions involving amino acids and reducing sugars during the drying and ripening stages. It is frequently reported in various cured and cooked meat products [48].

By comparing the concentration of aroma-active compounds, it is noteworthy that one compound identified by GC-MS in several ham samples might not necessarily be detected by panel assessors in all these samples. For example, 1-octanol (A7) was detected in BHCH, SXZH, and FZWH by GC-MS, with the highest concentration in SXZH compared to BHCH and FZWH. However, the GC-MS-O analysis revealed that 1-octanol (A7) was only detected in SXZH by the panel assessor. This discrepancy could be attributed to the detection threshold of aromas, which is the lowest concentration at which humans can perceive the aroma [69]. It is evident that the intensity of aroma-active compounds generally increased with their concentration, consistent with findings from previous studies [19].

Compounds with strong OI were considered more significant aroma-active compounds than those with middle or weak odor intensities. In BHCH, 17 aroma-active compounds (3 with strong OI, 10 with middle OI, and 4 with weak OI) were identified. Among them, 2-heptanol (A1), 1-hexanol (A2), and nonanal (B3) were identified as the most important, contributing to “woody/resinous”, “pungent”, and “sweet and popcorn” odors, respectively. Notably, 2-heptanol (A1), a secondary alcohol with woody and resinous aroma descriptors, was exclusively detected in BHCH and SXZH. These two hams, although differing in origins, are both considered premium products and share several processing attributes associated with high-end dry-cured ham production, such as long ripening periods (12–36 months), controlled cellar aging, and refined salting protocols. The presence of 2-heptanol (A1) may reflect advanced lipid oxidation and secondary alcohol formation, which are more likely to occur in well-matured hams due to prolonged exposure to enzymatic and oxidative processes [70].

In SXZH, 20 aroma-active compounds were identified, with 7 having strong OI, 12 with middle OI, and 1 with weak OI. SXZH had the highest number of both aroma-active compounds and those with strong OI among the five dry-cured hams, indicating a more complex odor profile. According to the odor evaluation, the “pungent” odor might be caused by the presence of 1-hexanol (A2); the “fruit, other than citrus” odor was linked to the presence of 2-nonanol (A10); the “sweet” and “popcorn” were mainly caused by nonanal (B3) and 2,6-dimethylpyrazine (H1); and the “woody/resinous” odor was associated with 2-heptanol (A1) and 2-nonanone (D2). Furthermore, one unknown aroma-active compound (UC5) contributed to the “wood/resinous” and “chemical” odor.

In FXZH, 11 aroma-active compounds were identified, including two with strong OI, five with middle OI, and four with weak OI. Among them, nonanal (B3) and 2,6-dimethylpyrazine (H1) were noted for their strong OI, with similar findings in SXZH. Despite 2,6-dimethylpyrazine (H1) being present in all five ham samples, it was perceived with strong intensity only in FXZH and SXZH, suggesting it may be a key compound distinguishing Xuanwei hams. For Jinhua hams (LTWH and FZWH), 23 aroma-active compounds (12 for LTWH and 18 for FZWH) were identified. Among them, 1-hexanol (A2), nonanal (B3), and heptanal (B17) were considered the more important aroma-active compounds, along with three unknown compounds (UC8, UC9, and UC10).

The hedonic assessment was conducted by the panelists to evaluate the relative pleasantness or unpleasantness of the aroma-active compounds, using a scale ranging from 1 (completely dislike) to 10 (very good, pleasant, and agreeable). Among the identified compounds, 1-octanol (A7), nonanal (B3), 3-octanone (D3), 5-ethyldihydro-2(3H)-Furanone (D4), dihydro-5-pentyl-2(3H)-furanone (D6), 5-butyldihydro-2(3H)-furanone (D8), 2-pentyl-furan (H2), and two unknown compounds (UC1 and UC10) all received scaled values higher than 6, suggesting they contribute positively to the sensory attributes of the dry-cured hams. Notably, 3-Octanone (D3) was unique to BHCH (the most expensive ham), while 1-octanol (A7) and 2-pentyl-furan (H2) were exclusive to SXZH (the second most expensive ham). These compounds might be key factors influencing the price differences among the five dry-cured hams.

In BHCH, 17 aroma-active compounds were detected, with 9 also found in SXZH, 6 in FXZH, 6 in LTWH, and 9 in FZWH. Despite SXZH and FZWH having similar odor compositions to BHCH, the latter showed a more favorable aroma profile as evaluated by the sensory panel. This phenomenon could be due to the adverse effect of unpleasant odors which were evaluated by the panelists. In the experiments, aroma-active compounds with scaled values lower than four were regarded as unpleasant odors. Table 5 shows that there were two unpleasant odors in BHCH, while SXZH and FZWH had five unpleasant odors. A high profile of unpleasant odors contributes to the off-odor in dry-cured hams, which is considered the main quality defect of dry-cured hams [71].

**Figure 6:**
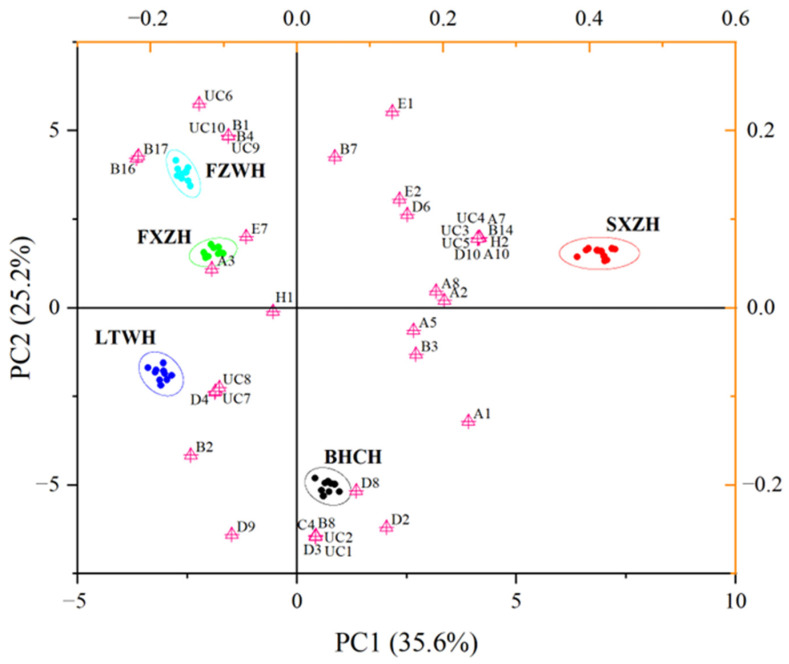
The PCA of the aroma-active compounds in the different dry-cured hams based on PC1 and PC2. (The dots with different colors represent the five different dry-cured hams; the ellipses with different colors represent the 95% confidence ellipse for the different hams; the triangles represent the aroma-active compounds.).

To further highlight the differences in aroma-active compounds among the five dry-cured hams, a PCA was performed using the hedonic assessment data. A PCA is a statistical tool used to intuitively show the data distribution in a low-dimensional space by extracting meaningful information from the raw data, allowing data to be grouped according to the similarity of the input characteristics to determine the distance between classes. The high separation between clusters indicates effective classification, while overlap suggests less distinction. The PCA extracted four principal components with eigenvalues greater than one, accounting for a cumulative contribution rate of 94.59%. The first component (PC1) and the second one (PC2) explained 35.6% and 25.2%, respectively, and the projections of these two primary components for the ham samples are shown in Figure 6. PC1, the most significant variable, was positively correlated with most aroma-active compounds with hedonic values higher than six (excluding one unknown compound, UC10). The two most expensive hams (BHCH and SXZH) were positioned on the positive PC1 axis, while the other three (FXZH, LTWH, and FZWH) were on the negative PC1 axis, indicating that PC1 can preliminarily distinguish between hams of different price points. These results suggest a positive correlation between aroma-active compounds with high hedonic scores and the price of dry-cured hams. Furthermore, the PCA showed clear separation among the five ham types, demonstrating the effectiveness of combining a PCA with a GC–O analysis to discriminate odor attributes. Notably, SXZH was located in the top right part of the plot, distinctly separated from the other clusters, reflecting its complex odor composition relative to the other hams.

### 3.3. E-Nose Analysis

**Figure 7:**
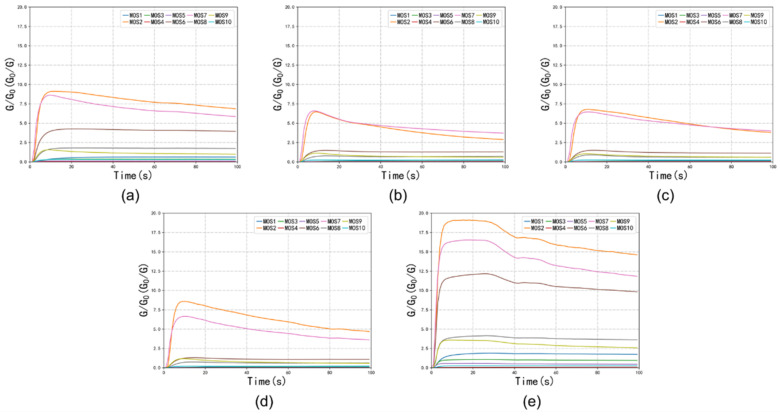
Examples of the 10 sensors’ response curves. (**a**) BMCH; (**b**) SXZH; (**c**) FXZH; (**d**) LTWH; (**e**) FZWH.

**Figure 8:**
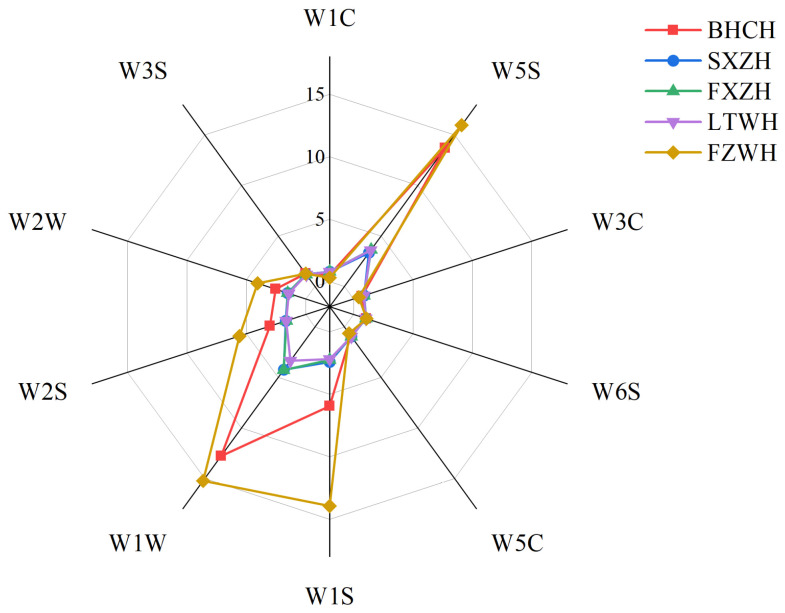
The radar chart of E-nose response data.

The E-nose was employed to differentiate the dry-cured hams and compare the results with those from the GC-MS-O analysis. The examples of the E-Nose response curves in the collection stage were shown in Figure 7. The data obtained from each sensor from 81-100 s was used as the stable value for the subsequent analysis. Figure 8 presents a radar chart depicting the responses (stable value) of the 10 sensors to the volatile compounds in the five ham samples. Sensors W1W, W1S, and W5S exhibited stronger responses to the volatile compounds in BHCH and FZWH compared to the other three samples, suggesting that these hams may contain higher levels of higher sulfur-containing compounds, terpenes, alkanes, and other volatile substances that triggered stronger sensor responses.

**Figure 9:**
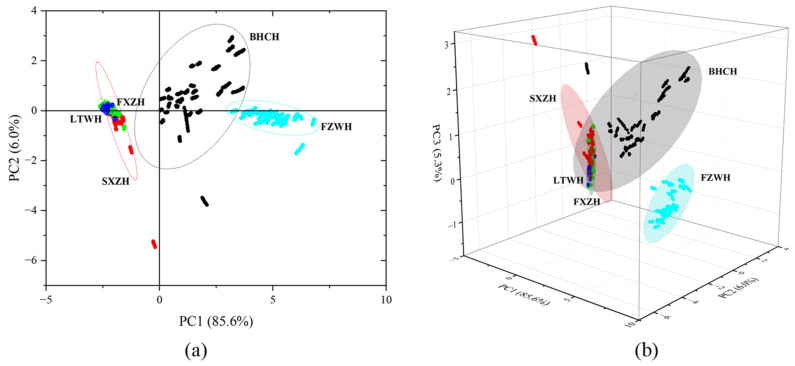
The PCA results of the of the ham samples by E-nose. (**a**) Projection of the first two principal components of the PCA; (**b**) projection of the first three principal components of the PCA. (The dots with different colors represent the five different dry-cured hams; the ellipses with different colors represent the 95% confidence ellipse for the different hams.).

To enhance the interpretation of the data, a PCA was performed on the stable values recorded by the 10 sensors for the ham samples. The PCA results are shown in Figure 9a. The x-axis represents PC1, which accounts for 85.6% of the variance, while the y-axis represents PC 2, which accounts for 6.0% of the variance. The cumulative variance explained by PC 1 and PC 2 was 91.6%, indicating minimal information loss. As shown in Figure 9a, the BHCH group (the most expensive ham sample) and FZWH group (the cheapest ham sample) were clearly distinguished from the other groups by the PCA analysis, while the other three ham groups (SXZH, FXZH, and LTWH) showed a relatively high overlap. Furthermore, the projections of the three primary components of PCA are shown in Figure 9b. The cumulative variance of PC 1, PC 2, and PC 3 was 96.3%, showing a smaller loss of information compared with the projections of the two primary components. However, the data distribution among the five ham samples remained similar, suggesting that while PCA combined with E-nose can effectively distinguish between the most and least expensive hams, it is less effective for distinguishing between all five dry-cured hams. The E-nose, therefore, serves as a quick analysis tool for broad classifications but does not provide the comprehensive and detailed evaluation achievable with a GC-MS-O analysis.

### 3.4. Correlation Between E-Nose and GC-MS-O

**Figure 10:**
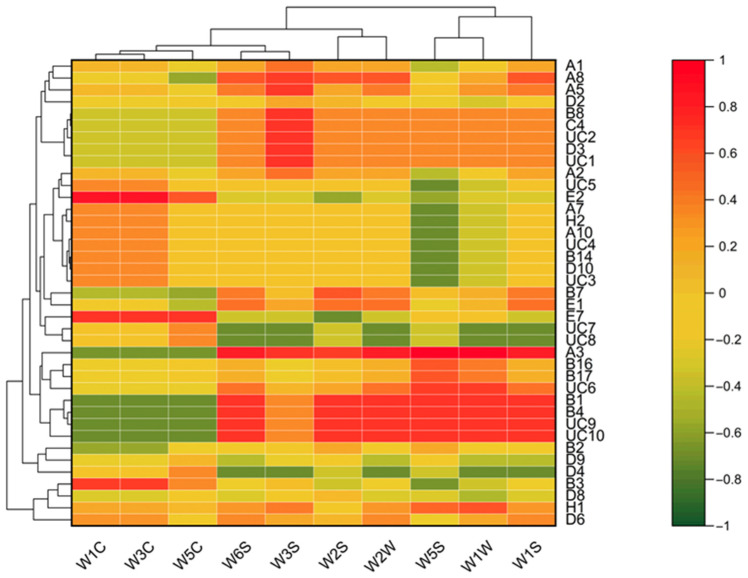
Correlation between aroma-active compounds and E-nose signals.

To explore the correlations between E-nose sensor responses and the aroma-active compounds identified by GC-MS-O, a correlation heatmap was constructed using the steady values from each of the ten E-nose sensors as the X variables, and the average hedonic scores of aroma-compounds as the Y variables. The resulting heatmap provides a clustering overview of sensor responses in relation to key odorants, facilitating the identification of compound–sensor patterns specific to each ham type. As shown in Figure 10, the 10 sensors can be grouped into different clusters based on their responses. For instance, a cluster of three sensors (W1C, W3C, and W5C) exhibited negative correlations with certain aroma-active compounds, including Hexanal (B1), Benzaldehyde (B4), Unknown compound 9 (UC9), and Unknown compound 10 (UC10). In contrast, the other seven sensors (W6S, W3S, W2S, W2W, W5S, W1W, and W1S) showed positive correlations with these compounds. According to the GC-MS-O results, these compounds were exclusively found in FZWH, indicating that the E-nose could effectively distinguish FZWH from other ham samples based on its specific responses to these aroma-active compounds. The findings align with the PCA results from the E-nose analysis. Additionally, the same sensor cluster (W1C, W3C, and W5C) exhibited negative correlations with 2-Nonenal, (E)- (B8), Pentadecane (C4), Unknown compound 2(UC2), 3-Octanone (D3), and Unknown compound 1 (UC1), while the other seven sensors correlated positively with these compounds. The GC-MS-O results indicated that these compounds were unique to BHCH, demonstrating that the E-nose could similarly differentiate BHCH from other ham samples.

The heatmap also shows that the 10 sensors have weak correlations with certain aroma-active compounds, such as 2-Nonanone (D2), 2(3H)-Furanone, dihydro-5-propyl- (D9), 5-Butyldihydro-2(3H)-furanone (D8), 2,6-Dimethylpyrazine (H1), and Dihydro-5-pentyl-2(3H)-furanone (D6). This weak correlation may account for the discrepancies between the GC-MS-O and E-Nose results. Among the 10 sensors, the W5S sensor displayed a relatively high correlation with the aroma-active compounds identified by GC-MS-O. However, this correlation was not sufficient for comprehensive analysis. Ultimately, the E-Nose is a chemical measurement system that assesses the chemical properties of sample gases rather than their odor characteristics [23]. A significant limitation of current E-Nose designs is the lack of specific odor sensors. To enhance the E-Nose’s performance and mimic biological olfactory systems more accurately, incorporating additional sensors that respond specifically to aroma-active compounds is essential.

## 4. Conclusions

This study comprehensively evaluated the quality characteristics of aroma-active compounds in various dry-cured hams using a combination of GC-MS-O and E-Nose techniques. A total of 29 aroma-active compounds were identified across the five types of dry-cured hams. Among these, nonanal, 5-butyldihydro-2(3H)-furanone, and 2,6-dimethylpyrazine were present in all five hams and contributed to the “sweet” and “popcorn” odors. The GC-MS-O results demonstrated that the intensity of these aroma-active compounds increased with their concentration, demonstrating their importance in sensory characteristics. Consumer acceptance can be adversely affected by off-odors, highlighting the impact of unpleasant odors on dry-cured hams. Additionally, the study highlighted the correlation between aroma-active compounds identified by GC-MS-O and E-Nose responses, offering insights into how the objective measurements of the E-Nose align with human sensory perception. The heatmap analysis demonstrated that certain sensors exhibited strong correlations with specific compounds, indicating that the E-nose could effectively distinguish ham types based on their specific aroma signatures. This research contributes to improving E-Nose systems by suggesting ways to better mimic biological olfactory structures. In addition, these findings provide a theoretical foundation for establishing aroma-based quality control standards and optimizing formulation strategies in dry-cured ham production Future advancements should focus on developing sensors with high sensitivity and selectivity based on the biological principles of olfaction (such as incorporating biosensors or receptor-mimicking materials) and combining these with advanced modeling techniques that map sensor outputs to sensory relevance, thereby enabling E-Nose systems to better replicate the specificity, perceptual accuracy, and diagnostic utility of the human olfactory function.

## Figures and Tables

**Table 1:** Details of the five ham samples analyzed in this study.

No.	Name	Category	Producing Area	Pig Breed	Price (USD/100 g)
1	BHCH	Bama Ham	Spain	Spain white pig	28.3
2	SXZH	Xuanwei Ham	Yunnan, China	Highland Wujin pig	22.0
3	FXZH	Xuanwei Ham	Yunnan, China	Highland Wujin pig	15.5
4	LTWH	Jinhua Ham	Zhejiang, China	Two-Ends-Black pig	6.1
5	FZWH	Jinhua Ham	Zhejiang, China	Free-range local pig	3.0

**Table 2:** Details of the 10 aroma groups.

NO.	Aroma Group	Main Characteristics
1	Floral	Aromatic, cologne, violet, subtle, rose, sweet
2	Woody/Resinous	Musty, strong, waxy, herbal, fatty, pungent, mushroom
3	Fruity, other than citrus	Sweet, strawberry, pineapple, banana, subtle, cucumber
4	Sickening	Putrid, sour milk, sweaty, excrement, strong, disgusting
5	Chemical	Anesthetic, cleaning fluid, medicine, paint, alcohol, pine oil
6	Minty	Cool, sweet, fennel, spice, medicine, aromatic
7	Sweet	Vanilla, caramel, chocolate, subtle, warm, malt
8	Popcorn	Burning, warm, strong, nutty, oil, peanut butter
9	Pungent	Onion, strong, household gas, burning, tartness, sulfide
10	Lemon	Citrus, sweet, cool, herbal, aromatic, subtle

**Table 3:** Details of the 10 sensors in the PEN3.

NO.	Sensor	Main Performance
1	W1C	Sensitive to aromatic compounds
2	W5S	High sensitivity to nitrogen oxides, broad range sensitivity
3	W3C	Sensitive to ammonia and aromatic compounds
4	W6S	Sensitive mainly to hydrogen
5	W5C	Sensitive to alkanes and aromatic components and less sensitive to polar compounds
6	W1S	Sensitive to methane, broad range sensitivity
7	W1W	Sensitive primarily to sulfur compounds and many terpenes and organic sulfur compounds
8	W2S	Sensitive to ethanol and less sensitive to aromatic compounds
9	W2W	Sensitive to aromatic compounds and organic sulfur compounds
10	W3S	Highly sensitive to alkanes

**Table 4:** The composition and concentration of volatile compounds identified in five different types of ham samples using HS-SPME-GC-MS.

No.	Compound	Retention Time (min)	CAS	Concentration (ng/g)				
				BHCH ^a^	SXZH ^b^	FXZH ^c^	LTWH ^d^	FZWH ^e^
**Alcohols**								
A1	2-Heptanol	9.96	543-49-7	1816.45 ± 5.39	3583.08 ± 1.67	n.d. ^f^	n.d.	n.d.
A2	1-Hexanol	8.61	111-27-3	1318.91 ± 4.45	2084.91 ± 2.75	n.d.	n.d.	1776.81 ± 0.64
A3	1-Octen-3-ol	13.58	3391-86-4	970.42 ± 3.49	n.d.	299.66 ± 1.57	n.d.	4414.67 ± 13.68
A4	1-Butanol, 3-methyl-	4.64	123-51-3	579.03 ± 2.34	1033.38 ± 2.55	n.d.	n.d.	122.27 ± 3.60
A5	1-Heptanol	13.07	111-70-6	512.55 ± 4.16	865.27 ± 2.94	334.38 ± 1.54	n.d.	359.18 ± 3.18
A6	1-Pentanol	5.35	71-41-0	373.15 ± 2.65	529.05 ± 3.72	n.d.	n.d.	634.3 ± 2.69
A7	1-Octanol	18.12	111-87-5	353.85 ± 2.68	702.11 ± 3.32	n.d.	n.d.	305.68 ± 3.27
A8	Phenylethyl Alcohol	20.12	60-12-8	337.77 ± 2.71	875.16 ± 2.92	60.91 ± 1.01	2.72 ± 0.36	137.56 ± 3.58
A9	Benzyl alcohol	16.15	100-51-6	157.62 ± 2.98	n.d.	n.d.	98.47 ± 0.58	118.45 ± 3.61
A10	2-Nonanol	14.66	628-99-9	n.d.	3128.18 ± 1.44	n.d.	n.d.	n.d.
A11	1-Tetradecanol	43.55	112-72-1	n.d.	215.9 ± 2.44	n.d.	n.d.	n.d.
A12	Cyclooctyl alcohol	17.95	696-71-9	n.d.	206.02 ± 2.46	n.d.	6.48 ± 0.36	n.d.
A13	1-Octanol, 3,7-dimethyl-	13.10	106-21-8	n.d.	n.d.	103.54 ± 0.98	n.d.	n.d.
A14	Ethanol	2.24	64-17-5	n.d.	n.d.	n.d.	n.d.	359.18 ± 3.18
A15	2-Octen-1-ol, (E)-	17.94	18409-17-1	n.d.	n.d.	n.d.	n.d.	217.8 ± 3.43
**Aldehydes**								
B1	Hexanal	6.16	66-25-1	7859.9 ± 19.90	n.d.	3310.9 ± 6.49	638.9 ± 0.67	13,283.48 ± 29.52
B2	Octanal	14.64	124-13-0	1704.94 ± 3.87	n.d.	n.d.	292.9 ± 0.50	2078.68 ± 0.10
B3	Nonanal	19.80	124-19-6	957.55 ± 3.51	1293.8 ± 1.52	356.3 ± 1.52	147.39 ± 0.56	1371.78 ± 1.37
B4	Benzaldehyde	12.50	100-52-7	669.11 ± 2.21	776.27 ± 3.15	539.02 ± 1.37	157.42 ± 0.56	1077.55 ± 1.89
B5	2-Heptenal, (E)-	12.34	18829-55-5	411.76 ± 2.6	n.d.	n.d.	n.d.	n.d.
B6	2-Octenal, (E)-	17.38	2548-87-0	286.3 ± 2.79	238.98 ± 2.91	133.38 ± 1.71	n.d.	n.d.
B7	Phenylacetaldehyde	16.59	122-78-1	41.54 ± 3.15	271.94 ± 4.31	60.79 ± 1.78	257.73 ± 0.51	133.73 ± 3.58
B8	2-Nonenal, (E)-	22.70	18829-56-6	196.51 ± 2.92	n.d.	10.13 ± 1.82	n.d.	80.81 ± 3.68
B9	2-Decenal, (E)-	30.65	3913-81-3	61.12 ± 3.12	365.88 ± 4.1	94.4 ± 0.99	n.d.	149.02 ± 3.55
B10	2,4-Nonadienal, (E,E)-	26.46	5910-87-2	24.66 ± 1.83	n.d.	n.d.	n.d.	80.24 ± 3.68
B11	2-Dodecenal, (E)-	37.67	20407-84-5	18.23 ± 1.83	n.d.	n.d.	1.67 ± 0.36	n.d.
B12	2,4-Decadienal, (E,E)-	35.10	25152-84-5	13.94 ± 1.86	n.d.	n.d.	n.d.	99.34 ± 3.64
B13	Decanal	25.85	112-31-2	11.79 ± 1.84	161.52 ± 2.88	n.d.	2.93 ± 0.36	22.92 ± 3.78
B14	Tridecanal	22.23	10486-19-8	n.d.	286.77 ± 4.28	n.d.	n.d.	n.d.
B15	Benzeneacetaldehyde, .alpha.-ethylidene-	31.05	4411-89-6	n.d.	227.44 ± 4.42	149.83 ± 1.70	n.d.	n.d.
B16	Butanal, 3-methyl-	3.36	590-86-3	n.d.	n.d.	1355.17 ± 1.67	505.52 ± 0.41	1180.72 ± 1.71
B17	Heptanal	9.98	111-71-7	n.d.	n.d.	612.11 ± 1.30	90.94 ± 0.59	1409.99 ± 1.30
B18	cis-4-Decenal	24.94	21662-9-9	n.d.	n.d.	n.d.	n.d.	103.17 ± 3.64
**Alkanes**								
C1	Heptane, 2,4-dimethyl-	3.89	2213-23-2	2940.23 ± 14.08	n.d.	n.d.	n.d.	n.d.
C2	Cyclopentane, butyl-	11.31	2040-95-1	128.67 ± 3.02	405.44 ± 4.01	n.d.	n.d.	n.d.
C3	Dodecane	25.54	112-40-3	96.5 ± 3.07	355.99 ± 4.12	115.11 ± 1.73	5.85 ± 0.36	84.06 ± 3.67
C4	Pentadecane	23.48	629-62-9	20.37 ± 1.86	n.d.	91.36 ± 1.75	n.d.	n.d.
C5	Tetradecane	39.64	629-59-4	13.94 ± 1.86	174.7 ± 2.51	169.93 ± 1.68	10.03 ± 0.62	31.84 ± 2.16
C6	Heptadecane	43.86	629-78-7	8.58 ± 1.85	192.83 ± 4.50	69.43 ± 1.77	n.d.	n.d.
C7	Hexane, 2,4-dimethyl-	6.18	589-43-5	n.d.	4748.34 ± 18.47	n.d.	n.d.	n.d.
C8	Tridecane	34.18	629-50-5	n.d.	257.1 ± 4.35	155.31 ± 1.69	4.6 ± 0.36	n.d.
C9	Heptane, 2,2,4,6,6-pentamethyl-	13.99	13475-82-6	n.d.	n.d.	518.92 ± 1.38	723.78 ± 0.31	n.d.
C10	Nonane, 2,2,4,4,6,8,8-heptamethyl-	15.84	4390-4-9	n.d.	n.d.	126.08 ± 1.72	n.d.	n.d.
C11	Hexadecane	46.79	544-76-3	n.d.	n.d.	91.36 ± 1.75	7.11 ± 0.36	n.d.
C12	Dodecane, 2,6,10-trimethyl-	38.50	3891-98-3	n.d.	n.d.	68.22 ± 1.00	2.09 ± 0.36	49.67 ± 3.73
C13	Heneicosane	46.78	629-94-7	n.d.	n.d.	n.d.	8.15 ± 0.62	106.99 ± 3.63
C14	Octadecane	43.84	593-45-3	n.d.	n.d.	n.d.	2.72 ± 0.36	72.6 ± 3.69
**Ketones**								
D1	2-Heptanone	9.37	110-43-0	1291.03 ± 3.03	979 ± 2.68	n.d.	n.d.	202.51 ± 3.46
D2	2-Nonanone	19.09	821-55-6	975.78 ± 3.48	1166.89 ± 2.24	n.d.	21.12 ± 0.35	n.d.
D3	3-Octanone	13.75	106-68-3	839.6 ± 1.95	n.d.	n.d.	n.d.	n.d.
D4	2(3H)-Furanone, 5-ethyldihydro-	16.94	695-6-7	79.87 ± 3.10	n.d.	21.09 ± 1.81	135.47 ± 0.57	n.d.
D5	2-Hexanone	5.83	591-78-6	270.21 ± 2.81	266.99 ± 4.33	n.d.	n.d.	n.d.
D6	Dihydro-5-pentyl-2(3H)-furanone	37.37	104-61-0	70.77 ± 3.11	326.33 ± 4.19	96.84 ± 1.74	13.59 ± 0.36	133.73 ± 3.58
D7	2-Decanone	24.81	693-54-9	50.4 ± 1.79	405.44 ± 4.01	n.d.	n.d.	n.d.
D8	5-Butyldihydro-2(3H)-furanone	29.71	104-50-7	47.18 ± 1.80	375.77 ± 4.07	102.32 ± 1.74	12.96 ± 0.36	30.56 ± 3.77
D9	2(3H)-Furanone, dihydro-5-propyl-	22.04	105-21-5	34.31 ± 1.81	n.d.	11.56 ± 1.82	15.02 ± 0.62	n.d.
D10	2-Tetradecanone	18.63	2345-27-9	n.d.	598.27 ± 3.56	n.d.	n.d.	n.d.
D11	Acetophenone	17.71	98-86-2	n.d.	187.88 ± 4.51	70.65 ± 1.00	n.d.	n.d.
D12	Amyl cyclopentenone	21.49	4819-67-4	n.d.	184.59 ± 2.49	n.d.	n.d.	n.d.
D13	3,5-Octadien-2-one	17.97	38284-27-4	n.d.	n.d.	184.55 ± 1.67	n.d.	n.d.
D14	3,5-Octadien-2-one, (E,E)-	19.18	30086-02-3	n.d.	n.d.	158.97 ± 1.69	n.d.	412.68 ± 3.08
D15	3-Octen-2-one	16.37	1669-44-9	n.d.	n.d.	n.d.	n.d.	431.78 ± 3.05
**Esters**								
E1	Octanoic acid, ethyl ester	25.17	106-32-1	n.d.	499.38 ± 3.79	116.94 ± 1.73	3.34 ± 0.36	82.79 ± 2.08
E2	Butanoic acid, 3-methyl-, ethyl ester	8.00	108-64-5	n.d.	370.83 ± 4.09	363.61 ± 1.52	n.d.	n.d.
E3	Heptanoic acid, ethyl ester	19.47	106-30-9	n.d.	234.04 ± 2.90	71.26 ± 1.77	n.d.	76.42 ± 3.68
E4	Nonanoic acid, ethyl ester	33.77	123-29-5	n.d.	217.55 ± 4.44	n.d.	n.d.	n.d.
E5	Acetic acid, hexyl ester	15.12	142-92-7	n.d.	174.7 ± 2.51	n.d.	n.d.	n.d.
E6	Decanoic acid, ethyl ester	39.36	110-38-3	n.d.	159.87 ± 2.54	126.08 ± 1.72	n.d.	58.59 ± 2.11
E7	Hexanoic acid, ethyl ester	14.43	123-66-0	n.d.	n.d.	1207.78 ± 0.79	n.d.	n.d.
**Acids**								
F1	Hexanoic acid	15.35	142-62-1	n.d.	n.d.	n.d.	311.09 ± 0.49	982.02 ± 2.06
F2	Acetic acid	2.67	64-19-7	n.d.	n.d.	n.d.	n.d.	371.92 ± 1.66
**Terpenes**								
G1	Naphthalene, 1,2,4a,5,8,8a-hexahydro-4,7-dimethyl-1-(1-methylethyl)-,[1S-(1.alpha.,4a.beta.,8a.alpha.)]-	44.45	523-47-7	n.d.	n.d.	69.43 ± 0.06	2.72 ± 0.36	n.d.
G2	D-Limonene	15.94	5989-27-5	n.d.	n.d.	n.d.	137.35 ± 0.57	393.57 ± 3.12
G3	Naphthalene, 1,2,3,5,6,8a-hexahydro-4,7-dimethyl-1-(1-methylethyl)-, (1S-cis)-	44.43	483-76-1	n.d.	n.d.	n.d.	n.d.	76.42 ± 3.68
**Others**								
H1	2,6-Dimethylpyrazine	10.44	108-50-9	160.84 ± 2.97	583.44 ± 3.59	484.21 ± 1.41	87.81 ± 0.59	197.42 ± 1.91
H2	Furan, 2-pentyl-	14.04	3777-69-3	n.d.	8534.15 ± 24.7	n.d.	n.d.	n.d.
H3	Pyrazine, tetramethyl-	18.84	1124-11-4	n.d.	n.d.	87.7 ± 1.75	n.d.	n.d.
H4	Dimethyl trisulfide	12.83	3658-80-8	n.d.	n.d.	75.52 ± 1.00	n.d.	n.d.

^a^ BHCH: Black Hoof Cured Ham. ^b^ SXZH: Superior-grade Xuan-Zi Ham. ^c^ FXZH: First-grade Xuan-Zi Ham. ^d^ LTWH: Liang-Tou-Wu Ham. ^e^ FZWH: Fei-Zhong-Wang Ham. ^f^ n.d. not detected.

**Table 5:** Odor-active compounds identified in the headspace of the five hams by GC–O.

No.	Quality		BHCH ^a^			SXZH ^b^			FXZH ^c^			LTWH ^d^			FZWH ^e^	
		DF ^f^	OI ^g^	HA ^h^	DF	OI	HA	DF	OI	HA	DF	OI	HA	DF	OI	HA
A1	Woody/Resinous	10	S ^i^	4.60 ± 0.52	10	S	5.20 ± 0.42									
A2	Pungent	10	S	1.80 ± 0.42	10	S	2.50 ± 0.53							10	S	1.50 ± 0.53
A3	Woody/Resinous	9	M ^j^	4.67 ± 0.50				9	M	4.22 ± 0.67				7	M	5.86 ± 0.38
A5	Woody/Resinous, pungent, chemical	7	M	5.43 ± 0.53	9	M	4.67 ± 0.50	10	W ^k^	3.50 ± 0.53				8	W	3.25 ± 0.71
A7	Popcorn				5	W	6.60 ± 0.55									
A8	Chemical	7	M	3.71 ± 0.49	4	M	4.75 ± 0.50							4	M	3.50 ± 0.58
A10	Woody/Resinous, fruit, other than citrus				10	S	5.70 ± 0.48									
B1	Lemon													7	W	5.71 ± 0.76
B2	Woody/Resinous, minty, chemical	9	M	5.67 ± 0.71							8	W	6.63 ± 0.52	10	M	4.70 ± 0.48
B3	Sweet, popcorn	10	S	7.30 ± 0.48	10	S	7.60 ± 0.52	10	S	7.30 ± 0.48	10	S	6.30 ± 0.48	10	S	6.20 ± 0.42
B4	Sweet, popcorn													9	W	4.56 ± 0.53
B7	Woody/Resinous				10	M	3.30 ± 0.48				9	M	2.67 ± 0.50	7	W	3.43 ± 0.53
B8	Woody/Resinous	9	M	4.89 ± 0.60												
B14	Woody/Resinous				8	M	5.38 ± 0.52									
B16	Woody/Resinous, chemical							9	M	4.22 ± 0.44	10	W	3.70 ± 0.48	7	W	4.57 ± 0.53
B17	Woody/Resinous, popcorn							10	M	5.30 ± 0.48	10	M	4.40 ± 0.70	10	S	5.60 ± 0.52
C4	Woody/Resinous	10	M	4.70 ± 0.48												
D2	Woody/Resinous	9	M	4.44 ± 0.53	10	S	3.60 ± 0.52				10	M	3.70 ± 0.48			
D3	Minty	10	W	6.50 ± 0.53												
D4	Woody/Resinous, sweet										10	W	7.60 ± 0.52			
D6	Sweet	10	W	7.40 ± 0.52	10	M	8.90 ± 0.57	9	W	7.44 ± 0.53				9	W	7.44 ± 0.53
D8	Sweet, popcorn, lemon	10	W	8.20 ± 0.42	10	M	8.00 ± 0.47	9	W	6.22 ± 0.44	10	W	8.50 ± 0.53	10	W	6.60 ± 0.52
D9	Sweet, popcorn	10	M	5.70 ± 0.48							9	W	8.33 ± 0.50			
D10	Woody/Resinous				10	M	5.10 ± 0.57									
H1	Popcorn	9	M	5.56 ± 0.53	10	S	5.10 ± 0.32	10	S	5.60 ± 0.52	10	M	5.00 ± 0.67	9	M	5.44 ± 0.53
H2	Lemon, minty				10	M	7.60 ± 0.52									
E1	Woody/Resinous				9	M	5.56 ± 0.53							7	W	5.43 ± 0.53
E2	Sickening, pungent				10	M	1.60 ± 0.52	10	M	1.60 ± 0.52						
E7	Fruity, other than citrus							6	W	4.50 ± 0.55						
UC1 ^l^	Minty	7	W	6.57 ± 0.53												
UC2	Popcorn	8	M	5.63 ± 0.52												
UC3	Woody/Resinous, chemical				10	M	4.60 ± 0.52									
UC4	Floral, woody/resinous				8	M	5.75 ± 0.46									
UC5	Woody/Resinous, chemical				10	S	3.40 ± 0.52									
UC6	Woody/Resinous							9	M	4.33 ± 0.50				9	M	5.11 ± 0.33
UC7	Minty, pungent										10	M	3.30 ± 0.48			
UC8	Pungent										10	S	1.50 ± 0.53			
UC9	Woody/Resinous													10	S	3.80 ± 0.42
UC10	Sweet													10	S	8.90 ± 0.57

^a^ BHCH: Black Hoof Cured Ham. ^b^ SXZH: Superior-grade Xuan-Zi Ham. ^c^ FXZH: First-grade Xuan-Zi Ham. ^d^ LTWH: Liang-Tou-Wu Ham. ^e^ FZWH: Fei-Zhong-Wang Ham. ^f^ DF: detection frequency. ^g^ OI: odor intensity. ^h^ HA: hedonic assessment, mean of ratings from ten assessors quantified on a scale from 1 (completely dislike) to 10 (very good, pleasant, and agreeable). ^i^ S: Strong. ^j^ M: Middle. ^k^ W: Weak. ^l^ UC: Unknown compound.

## Data Availability

The original contributions presented in the study are included in the article. Further inquiries can be directed to the corresponding author.

## Abbreviations

The following abbreviations are used in this manuscript:

GC-MS	Gas chromatography-mass spectrometry
E-Nose	Electronic nose
GC-MS-O	Gas chromatography-mass spectrometry-olfactometry
PCA	Principal component analysis
BHCH	Black Hoof Cured Ham
SXZH	Superior-grade Xuan-Zi Ham
FXZH	First-grade Xuan-Zi Ham
LTWH	Liang-Tou-Wu Ham
FZWH	Fei-Zhong-Wang Ham
HS-SPME	Headspace solid-phase microextraction
